# Image dataset acquired from an unmanned aerial vehicle over an experimental site within El Soldado estuary in Guaymas, Sonora, México

**DOI:** 10.1016/j.dib.2020.105425

**Published:** 2020-03-14

**Authors:** M. Sebastián Encinas-Lara, Luis A. Méndez-Barroso, Enrico A. Yépez

**Affiliations:** a Departamento de Ciencias del Agua y Medio Ambiente, Instituto Tecnológico de Sonora, 5 de Febrero 818 Sur, Cd. Obregon, Sonora 85000, Mexico; b Laboratorio Nacional de Resiliencia Costera, Sede Noroeste, 5 de Febrero 818 sur, Cd. Obregón, Sonora 85000, México; c Laboratorio Nacional de Geoquímica y Mineralogía, Sede Regional Sur de Sonora, 5 de Febrero, 818 Sur, Cd. Obregón, Sonora 85000, México

**Keywords:** Orthomosaics, Estuaries, Unmanned aerial vehicles, Aerial survey, Photogrammetry

## Abstract

It is well known that remote sensing is a series of procedures which detects physical characteristics of the earth surface by remotely-measuring its reflected and emitted radiation using cameras or sensors. Lately, the increasing use of unmanned aerial vehicles (UAVs) as remote sensing platforms and the development of small-size sensors have resulted in the expansion of continuous monitoring of earth surface at smaller spatial scales. For this reason, the integration of UAV- and consumer-grade cameras can be useful to acquire surface characteristics at plot or footprint scale. This dataset contains 314 aerial images covering an area of aproximately 18,800 m^2^ within the footprint of an Eddy covariance and meterorological station. The monitoring site was deployed at “El Soldado” estuary (27°57′14.4″ N and 110°58′19.2″ W) located in the southern coast of the Mexican State of Sonora. UAV flight path was programmed to flight in autonomous mode with an altitude of 30 m, a velocity of 5 m/s and a frontal and side overlap of 85 and 75% respectively. This dataset was created to support mapping surveys for surface classification and site description. This dataset is aimed to support researchers, stakeholders and general public interested in coastal areas, natural resources management and ecosystem conservation. Finally, this dataset could be also used for those interested in digital photogrammetry and 3D reconstruction as benchmark example to develop high resolution orthomosaics.

Specifications table

**Table:** 

Subject	Environmental Sciences, Computers in Earth Sciences
Specific subject area	Photogrammetry
Type of data	Image format: JPEG Geotagged Image format: EXIF format Image resolution: 4608 × 3456 pixels Image coordinate system: Lat/Long Image datum: WGS84
How data were acquired	Camera: MAPIR Survey2 RGB Aerial Platform: 3DR Solo Drone GCP measurement device: GNSS Trimble GeoXH 6000 Flight monitoring interface: Samsung Galaxy Tab A
Data format	Raw High quality images (Images with sharpness score lower than 0.5 were discarded) Geotagged Image coordinate system: Lat/Long Image size: 4 Mb Image composite color: RGB (Red, Green, Blue) Color depth: 24 bits
Parameters for data collection	Flight speed: 4 m/s Frontlap setting: 85% Sidelap setting: 75% Flight altitude: 30 m Camera focal aperture: f2.8 Camera ISO: 50 Shutter speed: 1/125 Camera interval shooting time: 2 s
Description of data collection	UAV flight path was programmed to fly in autonomous mode with an altitude of 30 m, a velocity of 5 m/s and a frontal and side overlap settings of 85 and 75% respectively. A total of 314 images were taken within the footprint of an Eddy covariance and meteorological station deployed at “El Soldado” estuary located in the southern coast of the Mexican State of Sonora. Coordinates were embedded in each image using the software GeoSetter v3.5 by extracting waypoints coordinates from the UAV flight controller log file.
Data source location	Institution: Instituto Tecnológico de Sonora City/Town/Region: Estero “El Soldado”, Guaymas, Sonora Country: México Latitude and longitude (and GPS coordinates) for collected samples/data: Latitude: 27.954000, Longitude: −110.972500]
Data accessibility	Repository name: Mendeley Data Data identification number: 10.17632/v6zcdkjzjd.1 Direct URL to data: https://data.mendeley.com/datasets/v6zcdkjzjd/1

## Value of the data

• Database is aimed to those interested in ecosystem conservation, natural resources management and studies in coastal ecosystems by applying digital photogrammetry and 3D reconstruction.
• Dataset can be used as a benchmark example to improve digital photogrammetry methods that helps to develop accurate high resolution orthomosaics (one photogrammetrically orthorectified image as product of mosaicking several images). However, several ground control points (GCP) are suggested to reduce geometric distortion.
• Dataset can be used as a mapping resource to identify objects of interest and to estimate the spatial extension of surface features in coastal environments.
• The dataset can be used as a baseline for a long-term monitoring schemes at El Soldado estuary. In addition, this dataset can be used for comparison in coastal landforms evolution with similar intertidal coastal environments in Northwestern Mexico or semiarid coasts in the world”.

## Data

**Fig. 1:**
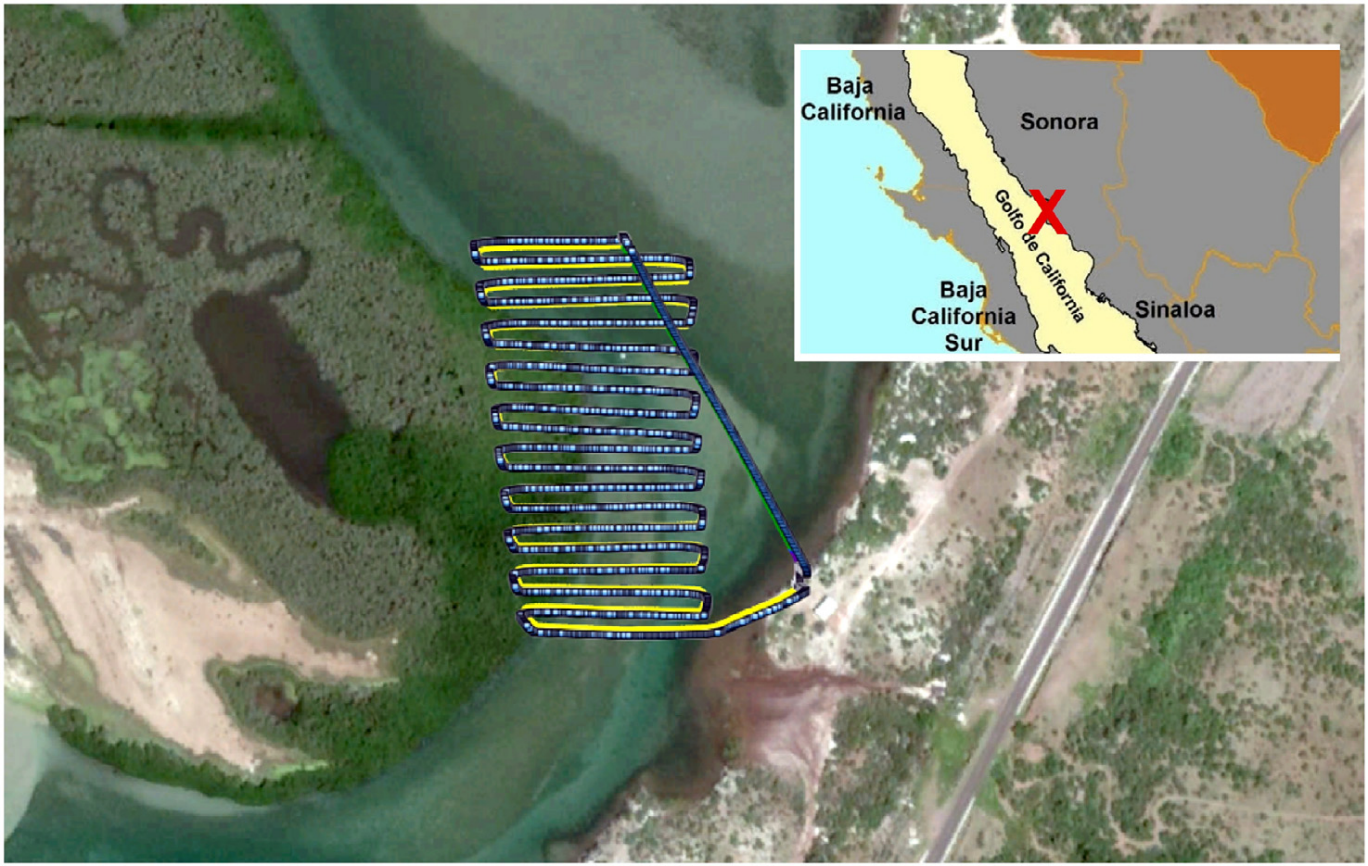
Sky view of the coastal strip surveyed within “El Soldado” estuary, in Guaymas, Sonora. The yellow line identifies the flight path followed by the UAV. The blue dots on the yellow line are the images taken using a commercial-grade digital camera. Upper-right figure shows the location of the study site within a regional domain in Northwestern, México.

This article describes a coastal survey routine by means of aerial images acquisition using an unmanned aerial vehicle and a commercial-grade RGB camera over an intertidal coastal strip within “El Soldado” estuary, in Guaymas, Sonora, Mexico (Fig. 1) [1]. 314 nadir-oriented images were collected using an unmanned aerial vehicle (UAV) over the footprint area of a eddy-covariance station deployed within El Soldado estuary, near Guaymas, México. UAV flew over the study site in autonomous mode with an altitude of 30 m, a velocity of 5 m/s and a frontal and side overlap settings of 85 and 75% respectively. Aerial images were taken using a commercial-grade digital camera using a focal aperture of f2.8, an ISO of 50, a shutter speed 1/125 and a shooting interval of 2 s. Images have a resolution of 4608 × 3456 pixels (4 M) with JPEG format. All images were analyzed and filtered based on sharpness quality. Only those images with a sharpness score higher than 0.5 were selected with potential use to perform a 3D reconstruction, ground survey and land surface evolution studies [2]. No ground control points (GCPs) were taken, however; all images were geotagged using UAV flight controller information than can help improving further 3D reconstruction or mosaicking [3]. Geotagging was embedded to all images in EXIF format.

## Experimental design, materials, and methods

This dataset contains 314 aerial photographs taken in March 02, 2019 at 9 a.m. local time (UTC - 16:00) over an experimental site instrumented with an eddy covariance tower and meteorological sensors at "El Soldado" estuary located at the South-eastern margin of the Gulf of California (27°57′14.4″ N and 110°58′19.2″ W). The images were taken with a 16 MP MAPIR Survey2 RGB camera pointed downwards at 90° with a field of view (FOV) of 82°, a shooting interval of two seconds, focal aperture of f/2.8 (default for MAPIR Survey2 Cameras), shutter speed set to 1/125 and ISO 50. Image quality was estimated based on image sharpness using the Agisoft Metashape's image quality tool. All images with a sharpness score lower than 0.5 were discarded [4] The camera was mounted on a commercial-grade Unmanned Aerial Vehicle (UAV, 3DR solo) to perform a 10-minute flight over the study site with an altitude of 30 m and a horizontal velocity of 4 m/s. Photographs sidelap was set to 75% while the frontlap was 85% in order to improve quality of the matching points and orthomosaic reconstruction [2, 4, 5]. Lock-copter orientation setting was activated to avoid sharp turns between waypoints [6]. Flight conditions during the aerial survey were clear sky with wind velocity less than 0.5 m/s. All images were geotagged using flight information extracted from the UAV flight controller using the software WinSCP v5.14.5 [7]. Controller´s log file was later converted to *.gpx format with the software Mission Planner v1.3.62 [8] and finally geotagged through the freeware GeoSetter v3.5 [9].

## Appendix Supplementary materials

Image, application 1
